# Case report: Percutaneous electrical neural field stimulation in two cases of sympathetically-mediated pain

**DOI:** 10.12688/f1000research.11494.1

**Published:** 2017-06-15

**Authors:** Lynn Fraser, Anna Woodbury

**Affiliations:** 1 Department of Anesthesiology, University of North Carolina, Chapel Hill, NC, 27599, USA; 2 Department of Anesthesiology, Veterans Affairs Medical Center, Atlanta, GA, 30033, USA; 3 Pain Medicine, Anesthesiology, Emory University School of Medicine, Atlanta, Georgia, USA

**Keywords:** percutaneous electrical neural stimulation; vagal stimulation; fibromyalgia; complex regional pain syndrome; central pain

## Abstract

**Background:** Fibromyalgia and complex regional pain syndrome (CRPS) are both chronic pain syndromes with pathophysiologic mechanisms related to autonomic nervous system dysregulation and central sensitization.  Both syndromes are considered difficult to treat with conventional pain therapies. **Case presentations:** Here we describe a female veteran with fibromyalgia and a male veteran with CRPS, both of whom failed multiple pharmacologic, physical and psychological therapies for pain, but responded to percutaneous electrical neural field stimulation (PENFS) targeted at the auricular branches of the cranial nerves. **Discussion:** While PENFS applied to the body has been previously described for treatment of localized pain, PENFS effects on cranial nerve branches of the ear is not well-known, particularly when used for regional and full-body pain syndromes such as those described here. PENFS of the ear is a minimally-invasive, non-pharmacologic therapy that could lead to improved quality of life and decreased reliance on medication. However, further research is needed to guide clinical application, particularly in complex pain patients.

## Introduction

Chronic pain syndromes encompass a poorly defined group of symptoms, including the chief perceptive state of ongoing pain. Patients often have comorbid findings, such as sleep disturbances, fatigue, headache, memory impairment, depression, anxiety, and bowel disturbance. All of these syndromes and associated symptoms can be precipitated by or worsened by stress and stressful life events, resulting in sympathetic nervous system activation ^1– 3^. Therefore, modulation of the sympathetic nerve system, for example through vagal nerve stimulation or sympathetic nervous blockade, may help in reducing pain symptoms. Chronic pain syndromes significantly impact function and quality of life for these patients.

Fibromyalgia is among these syndromes that pose a challenge to pain specialists. Perhaps the difficulty in treating these patients lies in the lack of insight into the cause of fibromyalgia. Although the exact mechanism remains unknown, a central sympathetically-mediated mechanism is suspected ^4– 6^. This central sensitization and pain-induced brain changes can be visualized with neuroimaging ^7^. Various psychological interventions for pain have therefore been employed to counter this sympathetic nervous system hyperactivity and central sensitization ^8^.

Fibromyalgia, as a chronic, multi-system illness that can present with a variety of symptoms, ranging from decreased physical activity, sleep disturbances, fatigue, emotional disorders, memory loss, and the hallmark musculoskeletal pain, has generated hypotheses from initial theories of muscle inflammation towards a theory of central nervous system derangement ^9^. In fact, using objective measures of activation of the sympathetic nervous system, Zamanuer *et al*. concluded that the degree of sympathetic activation was positively correlated with intensity of pain in fibromyalgia patients ^6^. From this data, the investigators postulated that sympatholytic drugs may lessen pain intensity in fibromyalgia. It has even been proposed that fibromyalgia and related disease entities be referred to as “Central Sensitivity Syndrome.” ^10^ Genetic, hormonal, psychosocial, and environmental factors may also play a role in this complex pathogenesis.

Similarly, complex regional pain syndrome (CRPS) is thought to have a mechanism of action involving dysfunction of the sympathetic nervous system, though its etiology is still poorly defined ^11^. It is believed there is a complex interplay between inflammatory mediators and the sympathetic nervous system, including sympathetic neurons releasing norepinephrine, which acts on adrenergic receptors, causing an increase in the amount of pro-inflammatory cytokines produced, ultimately leading to peripheral pain sensitization ^12^. Over time, maladaptive changes take place in the central nervous system, leading to central sensitization. While there is an increase in locally produced pro-inflammatory cytokines, interestingly, there is a lower level of norepinephrine measured in the affected limb ^13^. The sympathetic overdrive seen in CRPS is believed to be due to “sympathetic sprouting” and up-regulation of alpha-adrenergic receptors, leading to increased sensitivity to sympathetic nervous system neurotransmitters ^13^. Sympathetic blockade has been used in the treatment of CRPS, although it is unclear if this has long-term benefits ^14^.

The impact of fibromyalgia, CRPS, and other chronic pain syndromes is widespread. The difficultly in treating these syndromes imparts a financial burden on the healthcare system, with an unsurprising increased utilization of healthcare services. Mean total healthcare costs are estimated to be about three times higher among fibromyalgia patients when compared to age- and sex-matched patients without fibromyalgia ^15^.

We often find ourselves with limited tools in our armamentarium to effectively treat pain in this patient population. A multi-modal approach that includes non-pharmacologic therapy is desirable in addressing these elusive syndromes. Psychotherapy and physical therapy are considered fundamental in the treatment of fibromyalgia and CRPS, but are often insufficient as sole therapies to address the patient's symptomatology. Among the minimally invasive non-pharmacologic therapies employed in chronic pain therapy are acupuncture ^16^, transcutaneous electrical neural stimulation (TENS) ^17^, percutaneous electrical neural stimulation (PENS) ^18^, and more recently percutaneous electrical neural field stimulation (PENFS). Peri-auricular PENFS, which can be thought of as an evolution of auricular acupuncture and PENS to allow for stimulation of the entire ear, is thought to work by targeting the auricular branches of the cranial nerves. The auricular branches of the vagal nerve have previously been targeted for treatment of chronic pain syndromes ^19– 21^. Peri-auricular PENFS can plausibly exert a modulating effect on the central nervous system and on sympathetically-mediated pain through its access to auricular branches of the vagal nerve.

Here we present two cases of peri-auricular PENFS use in patients seen in our specialist pain medicine clinic at the Veterans Affairs Medical Center in Atlanta (GA, USA), with chronic pain syndromes refractory to multiple therapeutic interventions. The specific device used for PENFS in these patients was the Military Field Stimulator ^©^ (2016; Innovative Health Solutions INC.). Both patients gave written informed consent, in line with Declaration of Helsinki guidelines.

## Case presentation

### Case 1

A 56-year-old female veteran with a long-standing history of fibromyalgia and bipolar disorder, both diagnosed in 1990 following surgery for ovarian cancer, initially presented to the clinic on September 17, 2014 for interventional therapy for chronic low back pain of >10 years' duration. She reported receiving epidural steroid injections and sacroiliac joint injections in the past, from which she had temporary relief of her low back pain. In addition to her chief complaint of low back pain, she reported diffuse pain, in areas including the head, neck, arms, hands, hips, and buttocks. She was under the care of a specialist for fibromyalgia and taking multiple daily prescription medications to control the pain, including meloxicam, amantadine, topiramate, hydrocodone/acetaminophen, duloxetine, cyclobenzaprine, tizanidine, and gabapentin. Her bipolar disorder was treated with lamotrigine. She was taking indomethacin and sumatriptan on an as needed basis for headache. In addition, she was taking herbs and supplements, including maca, garcinia, L-lysine, vitamin E, vitamin D, coral calcium, and niacin. She was evaluated by a rheumatologist with inconclusive results regarding active inflammatory disease; she was previously on prednisone without reported benefit. Her daily function was significantly limited secondary to pain. She was unable to perform household chores and could not do physical activity for more than five minutes. If she over-exerted herself, she required one week without any physical activity. As a result, she reported poor quality of life. Her history was complicated by social issues, including strained relationships with her mother and daughter; she suffered a history of child abuse.

The patient agreed to try a series of acupuncture treatments. Pain scores were reported using the visual analog scale (VAS). After a series of acupuncture sessions between September 2014 and January 2015 (see Table 1), pain located in her head and neck was completely relieved, but the lower back pain persisted. She subsequently underwent a series of three peri-auricular PENFS applications ( Table 1: January 21, 2015; January 28, 2015, February 3, 2015), per manufacturer recommendations (see Discussion), for initial treatment of complex pain. Follow-up visits after the first application revealed 100% relief of diffuse pain that she attributed to fibromyalgia. She stated that she no longer required hydrocodone/acetaminophen. However, she noted her sacroiliac joint pain remained. The second application of the peri-auricular PENFS yielded similar results. After the third and final application, she returned to the clinic stating she had on-going complete relief of pain attributed to fibromyalgia. Although she reported persistent sacroiliac joint pain, she noted she was able to go golfing again without issues, which was a significant functional improvement from initial presentation, and something she had not been able to do in almost a decade. The sacroiliac joint pain was successfully treated with radiofrequency ablation of the L5 dorsal ramus and S1 and S2 lateral branches, as she had previously undergone sacroiliac joint injections with significant, but unsustained, relief from outside providers (series of 2 injections 1 month apart, multiple in 2014), and a repeated flurosocopy-guided injection in our procedure suite on January 9, 2015 yielded similar results.

**Table 1.** Pain procedures performed in case 1. VAS, visual analog scale.

Date	Treatment Type	Starting VAS	Ending VAS	Duration of relief
24-Sep-14	Acupuncture	8	0	10 days
8-Oct-14	Acupuncture	7	0	>7 days
15-Oct-14	Acupuncture	5	2	4 days
19-Nov-14	Acupuncture	6	0	3 weeks
7-Jan-15	Acupuncture	8	4	2 days
9-Jan-15	Bilateral sacroiliac joint injections	8	0	10 days
21-Jan-15	PENFS	10	2	>7 days
28-Jan-15	PENFS	5	2	>5 days
3-Feb-15	PENFS	3	2	~ 3 months
8-Jul-15	Acupuncture	10	5	7 days
31-Jul-15	Bilateral sacroiliac joint radiofrequency Ablation	10	2	7 months

Follow-up (February 9, 2015; March 20, 2015; July 8, 2015) revealed three months’ of >80% pain relief following peri-auricular PENFS treatment. The patient reported the return of diffuse pain coinciding with the death of her father, with whom she was close. She reported that her pain symptoms were exacerbated by anxiety and stress. At that time, a pain psychologist was included in her care. Acupuncture sessions were continued, and radiofrequency ablation of bilateral sacroiliac joints was continued. PENFS application was not repeated, (though recommendations from the manufacturer were that another one-time application could have resulted in alleviation of her symptoms again) as the patient felt that self-obtained cannabis oil had relieved the majority of her symptoms related to PTSD and pain. She felt that the device was bulky and difficult to wear, with adhesive that tended to entangle her hair, and preferred the combination of auricular acupuncture and cannabis oil for symptom management, despite decreased analgesic duration. The patient self-discontinued narcotic pain medications and pursued psychotherapy and auricular acupuncture, in combination with her own cannabis oil for the treatment of her symptoms.

### Case 2

A 52-year-old visually-impaired male veteran, with a history of left toe non-union fracture, presented for evaluation of worsening left lower extremity pain on December 17, 2014. According to the patient, he was previously diagnosed with CRPS > 10 years prior. The pain was characterized as left foot and leg pain radiating to the left hip accompanied by intense burning. These symptoms were previously controlled with a combination of NSAIDs, acetaminophen, and meditation, but he attributed an acute worsening of pain due to inability to meditate and the added burdens of becoming a primary caregiver for his mother. He recently tried gabapentin without benefit, and with side effects of sedation and balance difficulties. He had acupuncture in the past for myofascial pain in the upper neck and trapezius with good relief, and presented to the clinic requesting a series of acupuncture treatments for his uncontrolled left lower extremity pain. His medical history also included traumatic brain injury, chronic headaches, blindness, and “micro-seizures”, described as sensations of a shooting electrical and static electrical nature, all following a car accident with head impact.

Following his initial acupuncture treatment for CRPS on December 17, 2014, he had immediate pain relief on the day of therapy. On a follow-up visit, January 27, 2015, he endorsed >50% relief of the left lower extremity pain, as well as reduced intensity of the micro-seizures. At that time, the decision was made to begin a series of three placements of a peri-auricular PENFS device. Following the first application (January 27, 2015), he reported five days of >50% pain relief, accompanied by reduction of micro-seizures and an improvement in daily function. There was a noticeable increase in pain intensity when the battery life ended. He presented for a second application of the peri-auricular PENFS (February 3, 2015), after which he reported two days of 50% pain relief. After two days, he also reported return of micro-seizures that he noted seemed to correlate in intensity with left lower extremity pain. The final device application (February 9, 2015) resulted in two days of pain relief, at which time it came dislodged during visual evoked potential testing. He did state the needles stayed in place and seemed to give some relief despite not being connected to the stimulator.

After the final peri-auricular PENFS placement, the patient expressed a desire to continue with acupuncture sessions, which seemed to provide longer-lasting relief with each subsequent session. He felt that the device did not stay in place as well as the auricular acupuncture needles, and that it only gave him relief while it was in place. He also believed that the battery ran out quicker than it should have, so did not wish to try it again, particularly given the expense of the device relative to auricular acupuncture. Pregabalin was added to his medications and use of a TENS unit was used as an adjunct in an effort to achieve satisfactory pain relief, though he did self-discontinue the pregabalin due to side effects (balance difficulties), maintaining that ibuprofen and acetaminophen were better-tolerated and helped to alleviate his symptoms.

## Discussion

**Figure 1.**
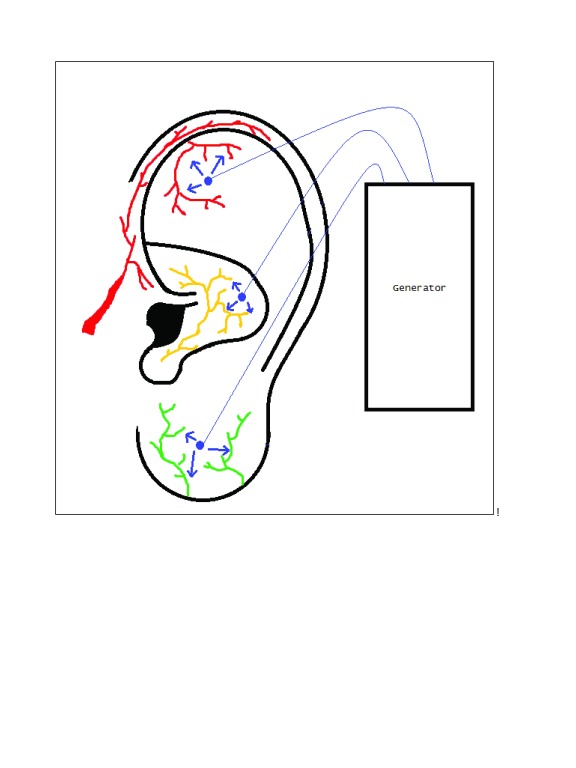
Example placement of percutaneous electrical neural field stimulation device. Electrode placement is provided in blue. Electrical field stimulation (blue arrows) of auricular branches of the trigeminal (red), vagus (yellow) and greater auricular (green) nerves is also depicted. Electrode placement can be varied to specifically target different points on the ear, though field stimulation provides broader coverage, regardless of placement.

The Military Field Stimulator is an FDA-approved nerve stimulator that is meant to target both acute and chronic pain by creating a field stimulation around the auricle to peripheral auricular branches of the cranial nerves, including the vagus, trigeminal, facial, hypoglossal, and occipital nerves. Placement is minimally invasive and involves three stimulating electrodes and a grounding electrode. Electrodes are placed percutaneously and secured in place ( Figure 1). A battery-operated generator is placed behind the ear that creates current that can be varied according to provider input. Stimulation is provided over a five-day period (120 hours) using a mild frequency between one and ten Hertz and amplitude of three Volts. A two-hour period of stimulation alternates with a two-hour period of rest for the treatment duration. Different treatment regimens exist, but for chronic pain, it is recommended that the patient have a series of three device placements, with each new trial occurring every seven days. The patient then has a rest period of seven days without the device. If pain increases during the rest period, the series of three device placements is repeated every seven days. Maximum analgesic benefit is achieved after six total placements.

The two patients described both obtained 50–100% relief of their chronic pain symptoms with utilization of peri-auricular PENFS. However, due to the discomfort associated with the device, both patients opted for alternate therapies (including auricular acupuncture) upon return of their symptoms. The patient in Case 1 achieved long-term (3 months' relief) following her PENFS series, until she experienced a life stressor. Manufacturer recommendations suggest that the device should be replaced for a five-day period should breakthrough pain occur after a period of analgesic effect. Had the patient chosen this option, it could have potentially restored her relief, but she had obtained cannabis oil from an unknown source, which she felt also gave her adequate relief of her pain. The patient in Case 2 could not complete the series, as the PENFS device became repeatedly dislodged. It is possible that he could have achieved longer-term relief, had he been able to complete the series in full. PENFS is distinct from manual acupuncture, electroacupuncture and TENS, although physiological effects may be related. In both Case 1 and Case 2, acupuncture had been performed prior to placement of peri-auricular PENFS, though results lasted for <30 days with each acupuncture treatment. It is possible that acupuncture may have primed these two patients for success with the PENFS device through neuro-modulatory mechanisms ^22, 23^.

At the time of this case report, there has been only one study published using a PENFS device in patients with persistent, non-malignant, chronic pain ^24^. In this study of 20 chronic pain patients with non-specific pain diagnoses, a significant decrease (average 65% improvement) in the VAS score was found after four treatments with the device. Though many studies have employed PENS at various body parts for localized pain, these studies did not use PENFS (which employs broader field stimulation), did not specifically target the cranial nerve branches in the ear, and did not employ PENFS/PENS for complex, widespread pain syndromes, such as CRPS or fibromyalgia. It is possible that the mechanism of action in which PENFS is applied to the ear can create a broader effect that alleviates not just regional, but full-body pain, perhaps through its action on the vagus nerve.

Stimulation of afferent nerves, particularly the vagus nerve, could modulate the autonomic nervous system, such that sympathetic or centrally-mediated chronic pain syndromes could benefit from the device ^6, 13, 19– 21^. Stimulation of the vagus nerve has been shown to inhibit spinal cord neurons below C3, but excite neurons between C1 and C3, suggesting that these areas may play a role in pain relief. Percutaneous stimulation of the auricular branches of the vagus nerve has been used to treat cervical dystonia ^25^. There is also evidence that stimulating the vagus nerve affects both the thalamus and hypothalamus, areas where pain modulation has been shown to occur ^26^.

The cases discussed here involve patients in which a specific FDA-approved PENFS unit, the Military Field Stimulator, was placed in an attempt to control chronic pain secondary to fibromyalgia and CRPS. Both patients had persistent pain despite initial treatment modalities and agreed to a trial of PENFS. Both patients arguably had some element of sympathetically-mediated pain, which may explain why field stimulation of the auricular branches of the cranial nerves, including the vagus (i.e. increasing parasympathetic nervous system stimulation) may have modulated their pain. Of note, the patient in Case 1 did not experience relief of her sacroiliac joint pain using the PENFS device. This may indicate that, while PENFS could be helpful in sympathetically-mediated pain states with central sensitization, it may not be as effective in arthritic pain.

As noted previously, vagus nerve stimulation has been shown to have effects on the thalamus and hypothalamus ^26^. In addition to the favorable impact on reported pain levels, the impact of vagal nerve stimulation on these brain segments is believed to counteract chemically induced nausea and vomiting due to the connection of the thalamus and hypothalamus to the “vomiting center” of the brain. Transcutaneous vagal nerve stimulation has also been shown to improve gastric motility ^27^. While further studies are needed to validate these initial findings, there appears to be promising possibilities for the use of PENFS devices or similar means of vagal nerve stimulations as adjuncts for multiple perceptive states.

This report describes an abbreviated trial of the PENFS device in two patients with sympathetically-mediated chronic pain syndromes. Further directions include recruiting a larger sample size and prolonged series of treatments (6 placements). However, based on our experiences with the Military Field Stimulator device, patients showed poor tolerance to repeated placement of the device due to its poor wearability, and thus showed a preference for alternative treatments, such as auricular and body acupuncture, despite the short-term duration of these treatments. A more wearable and user-friendly PENFS device is likely needed for increased patient compliance. It may be interesting to do a comparative study with PENFS and electroacupuncture or auricular acupuncture. Neuroimaging could also be employed to explore the neural mechanisms of PENFS effects in central pain states. A cost-benefit analysis should also be performed, as the Military Field Stimulator requires multiple applications with an indeterminate duration of relief. However, the costs of pharmacologic therapy and pain-related disability is also high. Well-designed, randomized controlled trials evaluating long-term pain and functional improvements related to PENFS use are needed.

Auricular PENFS is a promising therapeutic modality that requires further investigation. Further delineation of appropriate applications for peri-auricular PENFS is necessary to determine in which pain syndromes it would be most useful. It may be a useful adjunct in pain syndromes that have been otherwise difficult to control, but more studies are needed.

## Consent

Written informed consent for publication of their clinical details was obtained from the patients.
