# Supplementary material for: Case report: Percutaneous electrical neural field stimulation in two cases of sympathetically-mediated pain
Source: F1000Res. 2017 Jun 15;6:920. [Version 1] doi: 10.12688/f1000research.11494.1 (PMC5629543; doi:10.12688/f1000research.11494.1)
Supplement: Peer review history

# Peer review: Referee response for version 1

**DOI:** 10.5256/f1000research.12413.r23904

**Published:** 2017-10-04

**Authors:** Dalia Elmofty

**Affiliations:** 1 Department of Anesthesia & Critical Care, University of Chicago, Chicago, IL, USA

Stimulation of the auricular branch of the vagal nerve for the treatment of chronic pain has been reported in the literature. Percutaneous electrical neural field stimulation has been approved by the FDA and is classified as a minimal-risk device for the treatment of chronic pain. The exact mechanism is unknown, yet it is believed that the external ear contains branches of cranial nerves that project to brainstem nuclei involved in pain processing. Dr. Fraser and Woodbury provide an interesting description on the use of PEFNS for two cases of sympathetically-mediated pain.

 Editorial Comments

 Introduction:

1. The authors gave a detailed description on the pathophysiology of fibromyalgia and CRPS. The authors should consider providing a brief description on the mechanism of action of auricular stimulation.

Case 1:

1. The authors should consider stating that PENFS did not provide relief for pain from sacroiliitis and no need to further discuss other management (RFA).  Does the author have any data on objective improvement in pain after PENFS therapy for case 1 (fibromyalgia patient).

Case 2:

1. The authors should consider providing information regarding sympathetic-mediated symptoms and signs of CRPS for case 2 and if PENFS treatment reduced any of the sympathetic-mediated symptoms and signs.
2. The authors should consider providing a table for treatment modalities performed for case 2 and outcomes (as done for Case 1).

 

 Discussion:

1. The authors should consider discussing any potential side-effects from PENFS (exp. vaso-vagal response).
2. The authors should consider providing a more detailed explanation of the mechanism of action. This is a fairly new device.  There is evidence that it works on the NTS, RVM, hypothalamus, amygdale and spinal cord.

I have read this submission. I believe that I have an appropriate level of expertise to confirm that it is of an acceptable scientific standard, however I have significant reservations, as outlined above.

# Peer review: Referee response for version 1

**DOI:** 10.5256/f1000research.12413.r23509

**Published:** 2017-09-06

**Authors:** Albert Leung

**Affiliations:** 1 Department of Anesthesiology, University of California, San Diego, La Jolla, CA, USA

This is a very interesting case series documenting the efficacy PENFS in treating two complex chronic pain conditions. While the clinical outcome and potential related mechanisms are well described, it will be interesting for the reader if the authors can discuss the duration of pain relief from the treatment, which can vary from days to months as noted in table 1.

I have read this submission. I believe that I have an appropriate level of expertise to confirm that it is of an acceptable scientific standard, however I have significant reservations, as outlined above.

# Peer review: Referee response for version 1

**DOI:** 10.5256/f1000research.12413.r24043

**Published:** 2017-07-11

**Authors:** Katie Schenning, Andrei Sdrulla

**Affiliations:** 1 Department of Anesthesiology & Perioperative Medicine, Oregon Health & Science University, Portland, OR, USA

The case series describes the use of a Federal Drug Administration-approved electroacupuncture device, the Military Field Stimulator, which is a type of percutaneous electrical neural field stimulation (PENFS), for the treatment of patients with sympathetically mediated chronic pain syndromes. This is a novel approach to treat chronic pain, by stimulating auricular branches of cranial nerves and likely modulating and normalizing the autonomic systems in the brain. Overall, this is an interesting and well-written description of 2 patient cases.

**Introduction**

 The introduction describes well the complexity of fibromyalgia and complex regional pain syndrome (CRPS), including the hypotheses these syndromes are sympathetically mediated entities. However, we believe the introduction would be greatly improved by decreasing some of the details regarding fibromyalgia and CRPS, and instead focusing more on PENFS. For example, paragraphs two and three are repetitive and could be condensed into a single paragraph. On the other hand, PENFS is new technology and unfamiliar to most readers. The authors describe it later, in the Discussion section, however, the article would flow better if PENFS was introduced earlier. For example, Figure 1 could be part of the introduction.

**Case Presentation- Case 1**

 The first patient has fibromyalgia, as well as sacroiliac joint dysfunction and comorbid mood disorder. The patient in case one had a drastic, sustained improvement in her pain, as well as her functional status.

**Case Presentation- Case 2**

 For the patient with CRPS (case two) there is no mention of abnormal sympathetic tone in the affected extremity: swelling, edema, skin color changes, and temperature changes. This is important, as normalization of such findings (i.e. temperature difference between affected and unaffected leg during PENFS application) would support the notion that PENFS works by decreasing sympathetic tone, possibly in a similar way as a sympathetic block would act.

**Discussion**

 PENFS shows potential as a novel, non-pharmacological, low side-effect profile, treatment for patients with complex pain syndromes that are refractory to other therapies. The two cases responded well to this therapy, despite not using it as recommended by the manufacturer. Both patients had significant pain relief from acupuncture, before initiation of PENFS, and the authors mention that “… acupuncture might have primed these two patients for success with the PENFS device …”. The authors should also discuss the possibility that these patients responded to PENFS because they had good pain relief with acupuncture (selection bias). The authors should consider explaining the rationale for choosing these patients for PENFS trials. Was it because these patients had previously responded well to acupuncture? It may be that certain patients and possibly certain subtypes of fibromyalgia and CRPS respond to acupuncture, whereas others do not. The authors should also comment more broadly on the use of acupuncture for chronic pain syndromes in the discussion.

 Minor points:

- Remove **the** from “auricular branches of **the** cranial nerves” in abstract and discussion.
- “Zamanuer et al.” is misspelled.

We have read this submission. We believe that we have an appropriate level of expertise to confirm that it is of an acceptable scientific standard.
